# Ovarian lesions and tumors in infants and older children

**DOI:** 10.1515/iss-2021-0006

**Published:** 2021-08-11

**Authors:** Henning C. Fiegel, Stefan Gfroerer, Till-Martin Theilen, Florian Friedmacher, Udo Rolle

**Affiliations:** Department of Pediatric Surgery and Urology, University Hospital Frankfurt, Frankfurt, Germany; Department of Pediatric Surgery, Helios Berlin Buch, Berlin, Germany

**Keywords:** laparoscopy, ovarian cyst, ovarian torsion, ovarian tumors, surgery

## Abstract

**Objectives:**

Ovarian lesions are rare but frequent in children. Patients could present with abdominal pain, but ovarian lesions could also be incidentally found on ultrasound. Awareness is required in cases with acute, severe lower abdominal pain, as ovarian torsion could be the cause. Other lesions can be cysts or benign or malignant ovarian tumors. Thus, the aim of this paper is to review typical ovarian lesions according to age, imaging and laboratory findings, and surgical management.

**Methods:**

We retrospectively analysed the patient charts of 39 patients aged 10.4 ± 6.1 years (from 3 months to 18 years) with ovarian lesions treated in our institution between 01/2009 and 08/2020. All clinical and pathological findings of infants and children operated on for ovarian lesions were included.

**Results:**

Ovarian lesions in children younger than 2 years of age were typically ovarian cysts, and ovarian tumors were not observed in this age group. In older children over 10 years of age, tumors were more common – with mostly teratoma or other germ cell tumors, followed by epithelial tumors. Moreover, acute or chronic ovarian torsion was observed in all age groups. In general, ovarian tumors were much larger in size than ovarian cysts or twisted ovaries and eventually showed tumor marker expression of AFP or ß-HCG. Simple ovarian cysts or twisted ovaries were smaller in size. Surgery for all ovarian lesions should aim to preserve healthy ovarian tissue by performing partial ovariectomy.

**Conclusions:**

In adolescent girls with acute abdominal pain, immediate laparoscopy should be performed to rule out ovarian torsion. Careful imaging evaluation and the assessment of tumor markers should be performed in painless ovarian lesions to indicate an adequate surgical ovarian-sparing approach.

## Introduction

Ovarian lesions are not too rare in children. They could present with abdominal pain but could also be incidentally found on ultrasound. Awareness is required in cases with acute, severe lower abdominal pain, as ovarian torsion could be the cause. Other lesions can be cysts or benign or malignant ovarian tumors.

## Ovarian cysts

Cystic ovarian lesions are more common in infants and in older girls due to either maternal hormonal stimulation in newborns or either onset of hormonal activity in prepubertal age. Ovarian lesions in newborns and infants are mostly cystic in nature. Asymptomatic ovarian cysts are occasionally seen on ultrasound prenatally. The incidence of prenatal ovarian cysts is 1:2.500 [1]. Most of these cysts are (98%) smaller than 9 mm in diameter and usually spontaneously decrease over the first 6 months [2]. In newborns, cysts may present as palpable masses and can be diagnosed by ultrasound. Polycystic (stimulated) ovaries are very often – up to 98% – reported in female newborns. In the foetus, follicular stimulation is caused by maternal hormonal stimulation from oestrogen, placental HCG and foetal gonadotropins. After birth, there is a decrease in serum oestrogen and HCG from maternal hormones [3]. In addition, the hypothalamic-pituitary-ovarian axis is still immature, so gonadotropins may persist in the first few months up to 2–3 years of life [3]. In ultrasound, multiple small cysts up to 9 mm in diameter are typically observed in hormonally stimulated ovaries [4]. Cysts can be of any size and are usually simple or possess a daughter cyst or a single septation. Occasionally, there may be a sedimentation. Complex cysts are very rarely associated with a tumor and rather are the result of haemorrhage or torsion of the ovary in the newborn. In rare cases, cysts can perforate, cause ascites, may lead to compression of the ureter or other retroperitoneal organs, or may cause peritonitis when necrotic [5]. Because the risk of a malignant lesion in a cystic ovarian mass in the newborn is an exceptionally rare condition, surgery for ovarian cystic mass can be postponed, and cysts can be observed by ultrasound. No cases of malignant lesions in ovarian cysts or masses in newborns have been reported [3]. Cysts in neonates and infants can be observed if (i) the cyst is clearly from the ovary, (ii) the tumor markers AFP and ß-HCG are in age-related normal values or the AFP level shows a normal decrease, (iii) the cyst is not complex, and (iv) the patient is asymptomatic. Indications for surgery are enlargement of the cystic lesion or complications and over a period of 3–6 months persistence of large cysts with a diameter over 4–5 cm [3, 6]. Although the treatment of cysts larger than 5–7 cm is still controversial in respect of the risk of acute ovarian torsion, conservative treatment and close follow-up may be a safe option [7], [8], [9]. If surgery for ovarian cysts is indicated, several approaches can be performed including percutaneous aspiration, cyst fenestration or cyst resection, and laparoscopic or open operation. The preservation of ovarian tissue should be warranted.

## Ovarian torsion

Ovarian torsion is a rare condition in infants and children with an incidence of approximately 5 per 100.000 [10]; however, it should not be missed in differential diagnosis [11]. Although the percentage of children with ovarian torsion is not exactly known and a rare condition, the percentage is likely to be higher in older children and adolescent girls. The mean age of girls with ovarian torsion is 11–15 years of age [10, 12]. Symptoms of acute ovarian torsion can be acute, severe abdominal pain, nausea and vomiting. In patients with older torsion, also fever can be observed. The diagnosis of ovarian torsion may be challenging, because symptoms can also be found in ovarian lesions without torsion – and in other acute abdominal differential diagnosis in childhood. Most children with ovarian torsion have a pathology in the ovary or adnexa that can lead to twisting and ischaemia (benign cystic teratoma, haemorrhagic or follicular cyst, paratubal cyst, cystadenoma and hydrosalpinx) [13]. Ovarian torsion must always be considered in young girls with acute lower abdominal pain or a pelvic mass.

## Ovarian tumors

Ovarian tumors can be of benign or malignant nature. Benign ovarian neoplasms are often asymptomatic. Gynaecologic malignant tumors account for only 2% of all types of malignancies in children, and 60–70% of these are ovarian tumors [14]. Acute abdominal pain is the most frequent symptom and may be caused by acute ovarian torsion, tumor or cyst rupture, or haemorrhage. Chronic symptoms may be recurrent abdominal pain, anorexia, nausea and vomiting, which may be associated with occult ovarian torsion with necrosis or ovarian tumors [15]. Tumors can arise from the different tissues of the ovary: Germ cell tumors (yolk sac tumor, choriocarcinoma, immature or mature teratoma, seminoma/dysgerminoma, gonadoblastoma), Sex cord stromal tumors (juvenile granulosa cell tumor, Sertoli and or Leidig cell tumor, theca cell tumor), Epithelial tumors (serous or mucinous cystadenoma, borderline tumors, cystadenocarcinoma), or metastatic diseases and other malignancies (Hodgkin disease, neuroblastoma, wilmstumor). Ovarian lesions can be diagnosed by ultrasound examination. MRI should be preferred to CT scan because the diagnosis is very accurate and to minimize radiation exposure [16]. Laboratory examinations should include tumor markers of ovarian tumors, namely, AFP, ß-HCG and Ca-125. Mature teratomas in children and adolescent girls can be managed safely by ovarian-sparing resection when technically possible. The risk of recurrence or tumor spillage can be reduced by complete cyst/tumor resection, extracorporeal manipulation in the open surgical approach and intraoperative irrigation. Malignant transformation of mature teratomas is reported to occur rarely in up to 2% of patients. MRI scans typically show a cystic-solid tumor of the ovary with a fat pad sign suggesting ovarian teratoma. AFP can be elevated. Immature teratomas contain immature neuroepithelial tissue or possible microscopic foci of yolk-sac tumors in the mass. In cases of immature teratomas, the levels of AFP may be elevated. Because there is a higher risk of recurrent disease, immature teratomas may need adjuvant chemotherapy. Yolk-sac tumors, or endodermal sinus tumors, are the second most common type of pure tumor reported in the literature. Yolk-sac tumors are often associated with elevated AFP levels and are more aggressive tumors that often can metastasize within the perineum to the liver, lung or brain. Embryonal carcinoma and choriocarcinoma often show elevated levels of ß-HCG, and precious puberty is often reported. Both tissues are commonly seen as mixed lesions; thus, patients may have elevated levels of AFP, ß-HCG, or both. Stroma tumors of the sex cord are Sertoli cell tumors, Sertoli-Leydig cell tumors, granulosa cell tumors and others that arise from the stromal components of the ovary. They often produce hormones that may cause clinically apparent signs in patients. Granulosa cell tumors account for 1–10% of malignancies in girls and adolescents. Tumors in children and adolescents show a distinct behaviour compared to tumors in adults and thus are referred to as “juvenile granulosa cell tumors”. They often may be associated with pseudoprecocious puberty and galactorrhoea. Most of the lesions are limited to the ovaries, and the outcome from surgical resection is good. Sertoli-Leydig cell tumors, or arrhenoblastomas, are rare tumors that may cause elevation of serum testosterone consecutively, leading to signs of virilization. The tumors may have cystic and solid components. Epithelial tumors arise from the surface epithelium of the ovary and are rare in children. In children, epithelial tumors are most commonly serous and mucinous tumors. These tumor types may be benign, malignant or have low malignant potential (borderline tumors). Adenocarcinomas or cystadenocarcinomas are very rare in childhood and have a poor prognosis.

## Tumor markers

Alpha fetoprotein (AFP) and ß-human chorion gonadotropin (ß-HCG) are markers of tumorous lesions of the ovary [7]. AFP can be a marker of immature ovarian teratoma, yolk sac tumor, and embryonal carcinoma. ß-HCG can be a marker of malignant germ cell tumors, choriocarcinoma, and embryonal cell carcinomas. Of note AFP is physiologically elevated in newborns and infants for the first 9–10 months [17]. At that time, repeated assessment of AFP values with a timely decrease is mandatory for diagnostic evaluation.

## Imaging

Ovarian lesions can be diagnosed by ultrasound examination. MRI should be preferred to CT scan because the diagnosis is very accurate and to minimize radiation exposure [16]. Teratomas typically present in the MRI scans as a cystic-solid tumor of the ovary with a fat pad sign. AFP can be elevated. In the MRI scan, cystadenomas are usually very large cystic lesions of the ovary.

Thus, the aim of this paper is to review typical ovarian lesions according to age, imaging and laboratory findings, and surgical management.

## Materials and methods

### Patients

We retrospectively analysed the patient charts of 39 female patients aged 10.4 ± 6.1 years (from 3 months to 18 years) with ovarian lesions treated in our institution between 01/2009 and 08/2020. All clinical and pathological findings of infants and children operated on for ovarian lesions were included. From the patient charts, age, primary symptoms, sonographic findings, MRI or CT scan findings, and laboratory findings, including tumor markers (AFP, ß-HCG, Ca 19-9, Ca 125), were assessed. The operative procedure, intraoperative findings and pathological diagnosis were also retrieved from the patient charts. All retrospective clinical data were obtained after written informed consent was obtained from the parents, and the study was approved by the ethical council of the University Hospital Frankfurt (approval Nr. 25/16).

### Statistical analysis

SPSS for Windows (Version 14.0; SPSS Inc., Chicago, IL, USA) was used for statistical analysis. Cross table statistics were performed using the X^2^ test (for two variables). Significance statements refer to p-values of two-tailed tests that were less than 0.05.

## Results

**Figure 1:**
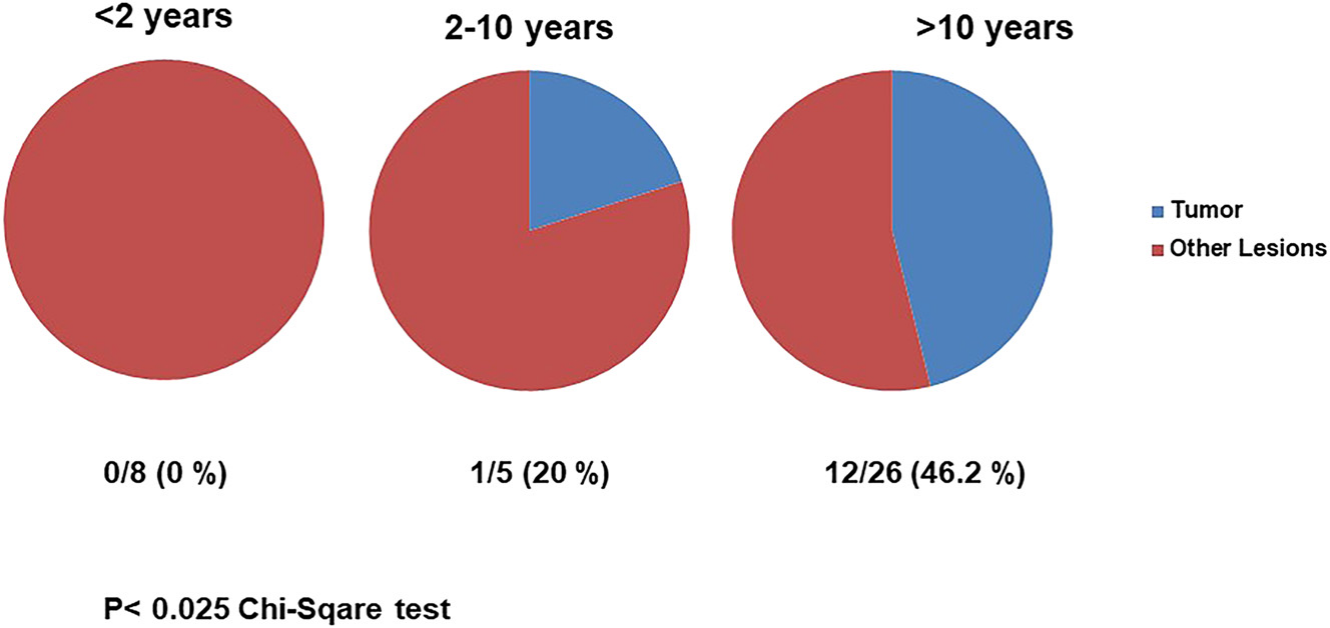
Graph of number of patients (percentage) with tumorous lesions in patients with ovarian mass according to age group. No occurrence of a tumorous lesions in patients with ovarian mass was observed in the age group younger than 2 years. An increasing portion of ovarian tumors were observed in the age groups from 2 to 10 years, and in females older than 10 years (n=39; p<0.025 by Chi-square test).

The data of 39 patients were available. The median age of the patients was 10.4 ± 6.1 years, with a range from 2.5 months to 18.1 years of age. The patients were grouped according to age as follows: age group <2 years, age group 2–10 years, and age group >10 years. A total of 8/39 (20.5%) patients were <2 years old, 5/39 (12.8%) patients were 2–10 years old, and 26/39 (66.6%) patients were >10 years of age. In total, 14/39 (35.9%) patients underwent laparoscopic surgery, 14/39 (35.9%) patients underwent combined laparoscopic and open surgery, and 11/39 (28.2%) patients underwent primary open surgery. In 20/39 (51.3%) patients, a total ovariectomy was performed, and in 8/39 (20.5%) patients, partial ovariectomy was performed. Other procedures included detorsion of the ovary (4/39 patients, 10.3%), cyst excision (4/39 patients; 10.3%), and cyst fenestration (2/39 patients; 5.2%). In 13/39 patients (33.3%), ovarian tumors were found. Tumors were in 6 cases mature teratoma, in 2 cases immature teratoma, yolk sac tumor in 1 case, Sertoli-cell tumor in 1 case, cystadenoma in 2 cases, and adenocarcinoma of the ovary in 1 case. In the age group <2 years, eight patients were identified; of these patients, 5/8 (62.5%) had ovarian torsion, 7/8 (87.5%) had an ovarian cyst, and no tumors were found in patients <2 years (Figure 1). In the 2–10 year age group, five patients were identified; of these patients, 3/5 (60.0%) had ovarian torsion, 1/5 (20.0%) had an ovarian cyst, and 1/5 (20.0%) had an ovarian tumor. In the >10-year age group, 26 patients were identified; of these patients, 10/26 (38.5%) had ovarian torsion, 7/26 (26.9%) had an ovarian cyst, and 12/26 (46.2%) had ovarian tumors. The increase in tumorous lesions in the older age groups and the higher proportion of ovarian cysts in the youngest age group were statistically significant (p=0.025; assessed by chi-square test).

Tumor size and longest tumor axis were assessed. In patients with tumors, the size (axis) was larger with a 15.9 ± 8.0 cm (n=13) longest axis measure compared to patients with a pure cystic lesion (longest axis 5.2 ± 1.4 cm; n=14) or patients with ovarian torsion (longest axis 6.2 ± 2.8 cm; n=18).

Expression of tumor markers are shown in Table 1. AFP was elevated in 5/39 patients (12.8%): two patients had an immature teratoma, one patients had a yolk sac tumor, one patient had a sertoli-cell tumor, and one patient had ovarian necrosis, respectively. ß-HCG was elevated in 2/39 patients (5.1%): one patient had an immature teratoma, one patient had a yolk-sac tumor. Ca 125 was elevated in 2/39 patients (5.1%): one patient had an mature teratoma, one patient had a sertoli-cell tumor. Ca 19-9 was elevated in 2/39 patients (5.1%): the two patients had a mature teratoma. On the other hand, no elevated tumor markers were observed in three patients with mature teratoma, two patients with cystadenoma, and one patient with an adeno-carcinoma.

**Table 1:** Table of tumor marker expression of different ovarian tumors.

Tumor marker	Marker expression in tumor entities
AFP (n=5)	Immature teratoma (2), yolk sac (1), sertoli-cell (1), necrosis (1)
ß-HCG (n=2)	Immature teratoma (1), yolk sac (1)
Ca 125 (n=2)	Mature teratoma (1), sertoli-cell (1)
Ca 19-9 (n=2)	Mature teratoma (2)
No tumor markers	Mature teratoma (3), cystadenoma (2), Adeno-Ca (1)

## Discussion

### Lesions in newborns and infants – cystic lesions

**Figure 2a–c:**
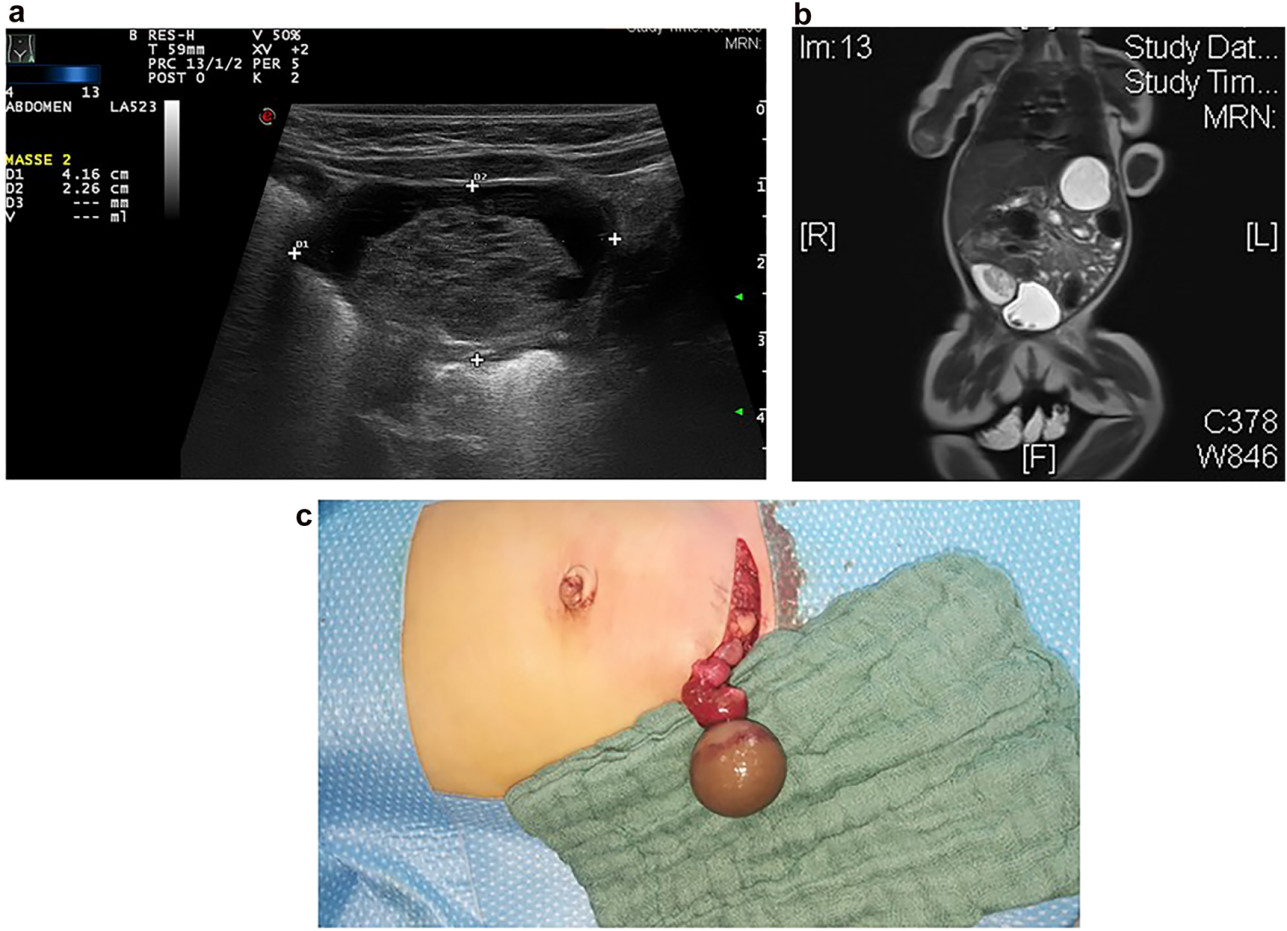
Sonography (a) and MRI (b) findings showed a cystic mass in a 6 week old infant with hemorrhagic solid mass and fibrous septation of the cystic mass typically suggesting ovarian necrosis. Intraoperative situs (c) revealed an old (intrauterine) ovarian torsion with a necrotic ovary.

In our own patient series, we observed no tumorous ovarian lesions in children younger than 2 years of age, but an increasing number of ovarian tumors in older children (Figure 1). This is in concordance with the literature, where no cases of malignant lesions in ovarian cysts or masses in newborns have been reported [3]. Ovarian lesions in children younger than 2 years are typically ovarian cysts, and ovarian tumors were not observed in this age group. In older children over 10 years of age, tumors were more common – with mostly teratoma or other germ cell tumors, followed by epithelial tumors. Moreover, acute or old ovarian torsion was observed in all age groups. Complex cysts in newborns are very often old (intrauterine) ovarian torsions due to large ovarian cysts (Figure 2a–c). Complex cysts are very rarely associated with a tumor and rather are the result of haemorrhage or torsion of the ovary in the newborn [5]. Because the risk of a malignant lesion in a cystic ovarian mass in the newborn is an exceptionally rare condition, surgery for ovarian cystic mass can be postponed, and cysts can be observed by ultrasound.

### Lesions in older children – ovarian torsion

**Figure 3a/b:**
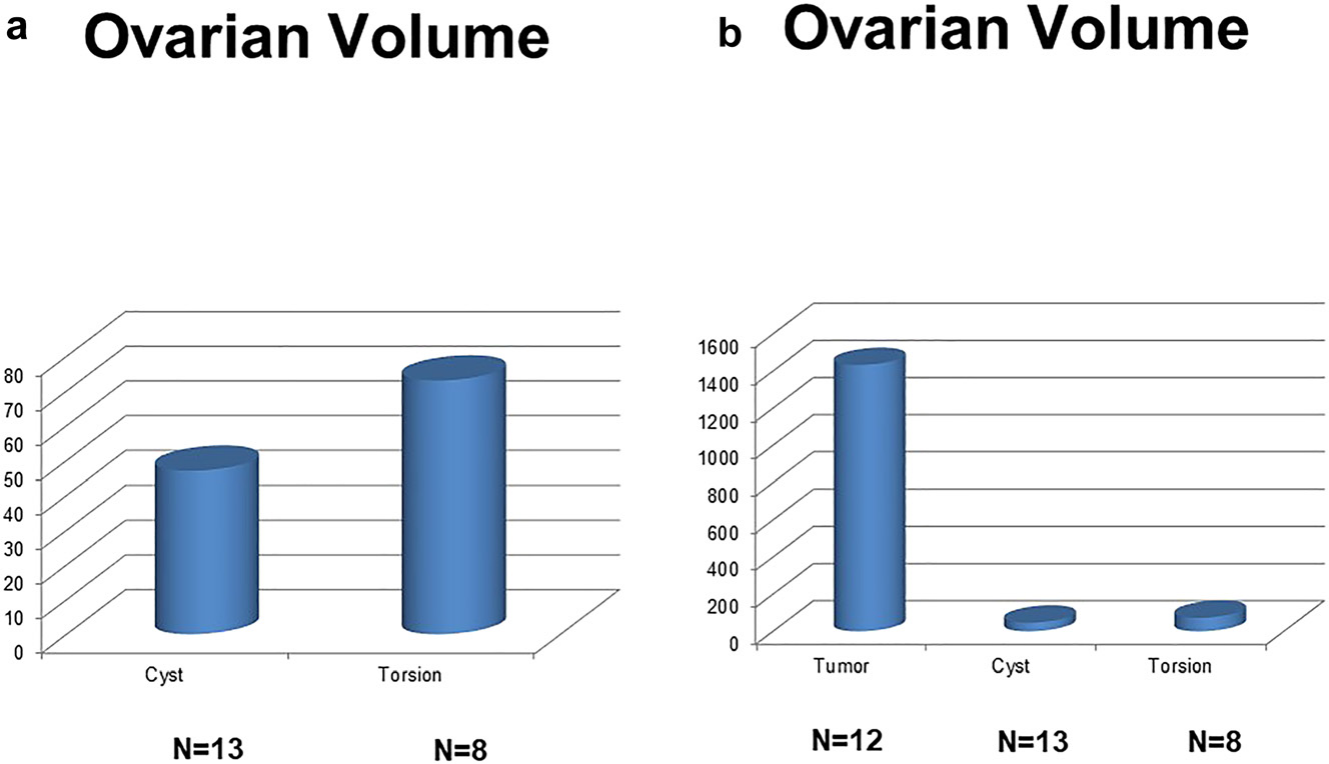
Graph ovarian volume of ovarian cysts and torsed ovaries (a) showed an increased volume in ovary torsion. Graph average ovarian mass volume showing that tumorous ovarian lesions (b) showed a much higher volume when compared to ovarian cysts or ovaries with torsions.

**Figure 4:**
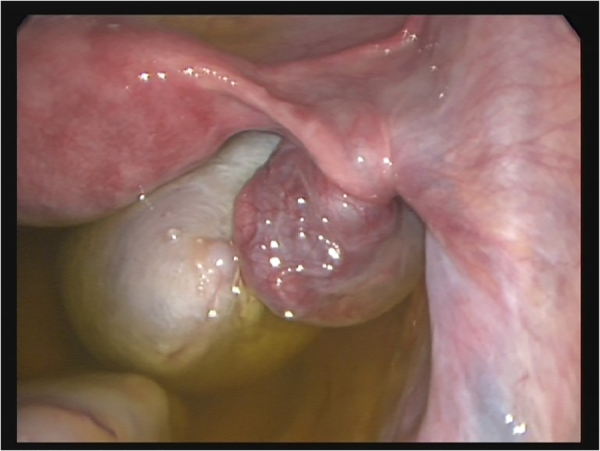
Intra-OP picture of ovarian torsion with vital ovary. Laparoscopic detorsion is warranted to preserve the ovary timely.

Mostly, the volume of torsed ovaries was greater than that of simple ovarian cysts or normal ovaries (Figure 3a). In adolescent girls with acute abdominal pain and enlarged ovaries on ultrasound, immediate laparoscopy should be performed to rule out ovarian torsion (Figure 4). Most children with ovarian torsion have a pathology in the ovary or adnexa that can lead to twisting and ischaemia, which could also lead to increased ovarian volume (benign cystic teratoma, haemorrhagic or follicular cyst, paratubal cyst, cystadenoma and hydrosalpinx) [13]. Ovarian torsion must always be considered in young girls with acute lower abdominal pain or a pelvic mass. Immediate laparoscopy should be performed to rule out ovarian torsion. Recently, the importance of immediate diagnosis and surgery in preadolescent patients was highlighted since in cases of benign lesions with torsion, ovarian preserving surgery should be the aim [18].

### Lesions in adolescents – ovarian tumors

**Figure 5a/b:**
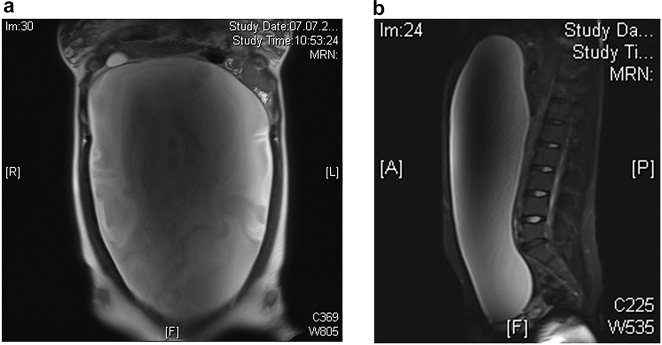
MRI scan showed a big monocystic tumor which fills nearly the whole abdominal cavity (a sagittal; b lateral). After complete resection, histological examination revealed a serous cystadenoma of the ovary.

In adolescent girls, benign or malignant ovarian tumors were more frequent than in younger girls or infants (Figure 1). Careful imaging evaluation and tumor marker assessment should be performed in painless ovarian lesions to indicate an adequate surgical ovarian sparing approach. Ovarian lesions can be diagnosed by ultrasound examination. MRI should be preferred to CT scan because the diagnosis is very accurate and to minimize radiation exposure [16]. The volume of tumorous ovarian lesions was much larger than that of other ovarian lesions, such as cysts or torsion (Figure 3b). This is in concordance to the literature. Mature teratomas typically show in MRI scans a cystic-solid tumor of the ovary with a fat pad sign suggesting ovarian teratoma. AFP can be elevated [19]. Epithelial tumors are most commonly cystadenomas, which arise from the surface epithelium of the ovary and are rare in children. In the MRI scan, cystadenomas were usually very large cystic lesions of the ovary [19] (Figure 5a/b).

**Figure 6a–c:**
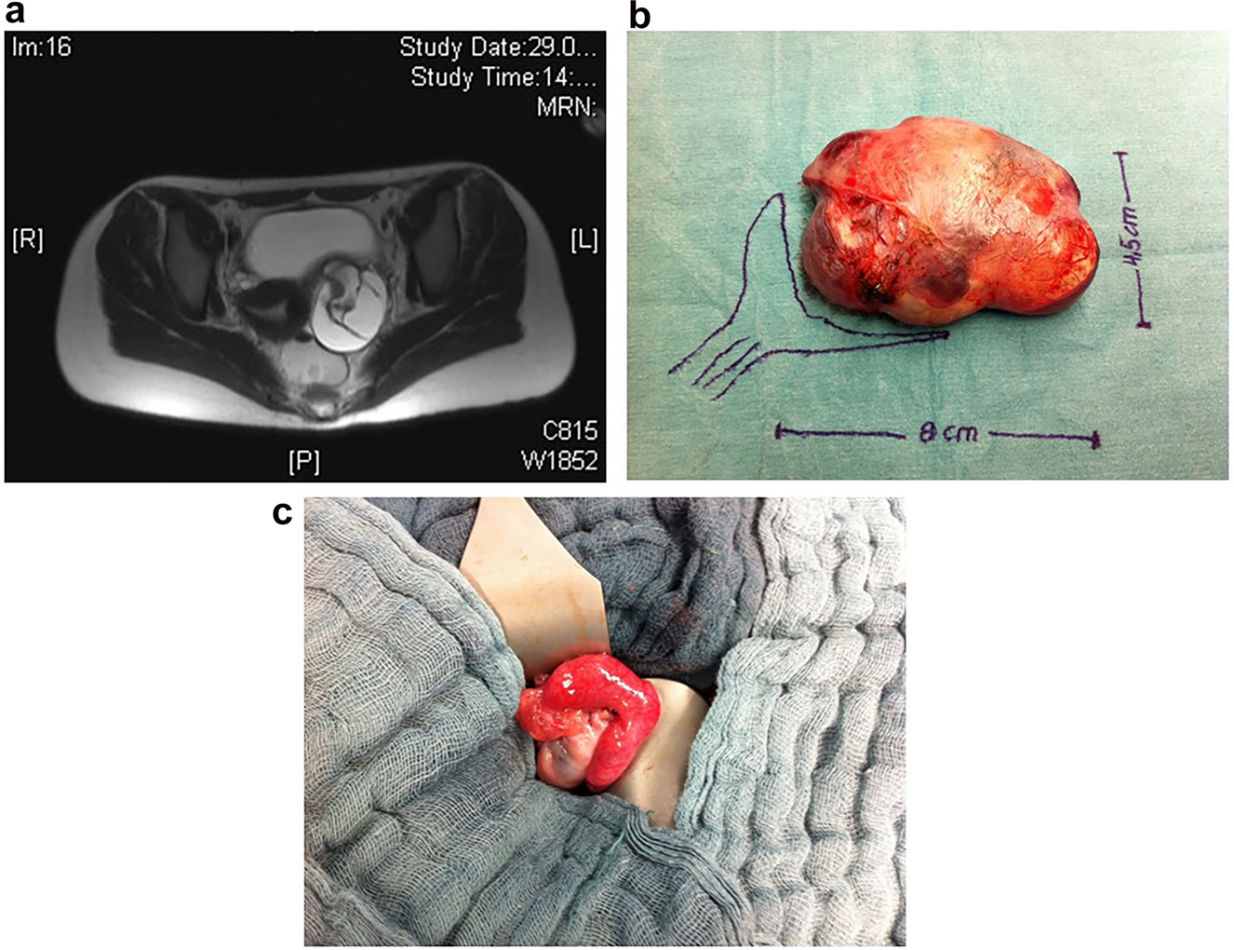
MRI scan (a) showed a big cystic-solid tumor of the ovary suggesting ovarian teratoma. AFP values were normal. *Ex-situ* photograph of the opened resected tumor showed the typical appearance of mucous and hairy contents of a mature teratoma (b). Intra-OP picture showed partial resection of the ovarian mass, preserving healthy ovarian tissue (c).

Laboratory examinations should include tumor markers of ovarian tumors, namely, AFP, ß-HCG and Ca-125 (Table 1). In our patients, we found the expression of AFP in 12.8% and ß-HCG in 5% of the patients. In the literature, the value of preoperative assessment of tumor markers was recently highlighted, with a strong recommendation to assess the whole panel of tumor markers and LDH [20]. In a recent report, good outcomes was reported for ovarian sparing tumor resection in children and adolescent girls [21]. The risk of recurrence or tumor spillage can be reduced by complete cyst/tumor resection, extracorporeal manipulation in the open surgical approach and intraoperative irrigation. Ovarian-sparing resection should be the surgical approach for mature teratomas of the ovary (Figure 6a–c).

## Conclusion

Ovarian lesions in newborns and infants are typically ovarian cysts, and ovarian tumors were not observed in this age group. In older children and adolescents, tumors were more common – with mostly teratoma or other germ cell tumors, followed by epithelial tumors. In general, ovarian tumors were much larger in size and eventually showed expression of tumor markers AFP or ß-HCG. Simple ovarian cysts or twisted ovaries were smaller in size. Surgery for mature ovarian teratoma should aim to preserve healthy ovarian tissue by partial ovariectomy. Moreover, acute or old ovarian torsion was observed in all age groups. In adolescent girls with acute abdominal pain, immediate laparoscopy should be performed to rule out ovarian torsion.

## Supplementary Material

Supplementary Material
