# Supplementary material for: Ovarian lesions and tumors in infants and older children
Source: Innov Surg Sci. 2021 Aug 11;6(4):173–9. doi: 10.1515/iss-2021-0006 (PMC9294339; doi:10.1515/iss-2021-0006)
Supplement: Supplementary file 1 — Supplementary Material [file j_iss-2020-0006_suppl.pdf]

Reviewer Assessment

H Fiegel, et al.: Ovarian Lesions and Tumours in Infants and Older Children

Reviewers' Comments to Original Submission

Reviewer 1: Shehata, Sameh

Date received: 05-Mar-2021

Reviewer recommendation: **Return to author for major modifications**

Reviewer overall scoring: **Medium**

Assessment Form scores: 5 = High/Yes; 3 = Medium/Adequate; 1 = Low

Is the subject area appropriate for the journal				2	
Does the title clearly reflect the paper's content?			3		
Does the abstract clearly reflect the paper's content			3		
Do the keywords clearly reflect the paper's content?		4			
Does the introduction present the problem clearly?					1
Are the results/ conclusions justified?			3		
How comprehensive and up-to-date is the subject matter presented?			3		
How adequate is the data presentation?					
Are units and terminology used correctly?			3		
Is the number of cases adequate?		4			
Are the experimental methods/ clinical studies adequate?			3		
Is the length appropriate in relation to the content?		4			
Does the reader get new insights from the article?				2	
Please rate the practical significance.			3		
Please rate the accuracy of methods.			3		
Please rate the statistical evaluation and quality control.				2	
Please rate the appropriateness of the figures and tables.		4			
Please rate the appropriateness of the references.				2	
Please evaluate the writing style and use of language.		4			
Please judge the overall scientific quality of the manuscript.			3		
Are the methods used worthy of reproduction in greater deal?	Yes				
Would you be willing to review a revision of this manuscript?	Yes				

Comments to author: A retrospective review of ovarian masses in children from a single institution. The introduction is very brief; it should include a recent review of relevant literature. The discussion is mainly a literature review (normally should be in introduction), the discussion should comment on the authors' results with a comparison of similar studies from other

centers. The conclusion should be based on the author's own results. The term " rare but frequent " is contradictory. Most of the references are old.

Reviewer 2: Kutasy, Balazs

Date received: 28-May-2021
Reviewer recommendation: **Accept in present form**
Reviewer overall scoring: **Excellent**

Assessment Form scores: 5 = High/Yes; 3 = Medium/Adequate; 1 = Low

Is the subject area appropriate for the journal	5				
Does the title clearly reflect the paper's content?	5				
Does the abstract clearly reflect the paper's content	5				
Do the keywords clearly reflect the paper's content?	5				
Does the introduction present the problem clearly?	5				
Are the results/ conclusions justified?	5				
How comprehensive and up-to-date is the subject matter presented?	5				
How adequate is the data presentation?	5				
Are units and terminology used correctly?	5				
Is the number of cases adequate?	5				
Are the experimental methods/ clinical studies adequate?		4			
Is the length appropriate in relation to the content?		4			
Does the reader get new insights from the article?		4			
Please rate the practical significance.	5				
Please rate the accuracy of methods.	5				
Please rate the statistical evaluation and quality control.		4			
Please rate the appropriateness of the figures and tables.		4			
Please rate the appropriateness of the references.		4			
Please evaluate the writing style and use of language.	5				
Please judge the overall scientific quality of the manuscript.	5				
Are the methods used worthy of reproduction in greater deal?	Yes				
Would you be willing to review a revision of this manuscript?	Yes				

Comments to author: It is a nicely written manuscript. Only one minor comment. One of the (main) indication of surgery in newborn girls with a large asymptomatic cyst is to minimise the risk of ovarial torsion.

Authors' Response to Reviewer Comments

Date received: 04-Jun-2021

Response to reviewer 1

1. „the introduction is very brief; it should include a recent review of relevant literature."
2. „the discussion is mainly a literature review (normally should be in introduction), the discussion should comment on the authors results with comparision of similar studies from other centers"

Reply & 2: We extensively reworked the introduction part of the manuscript transferringt the literature review from the discussion part to the introduction as suggested by the reviewers comments (see manuscript introduction part pp. 3 to 7– changes marked in red – paragraphs „ovarian cysts", „ovarian torsions", „ovarian tumors", „tumor markers" , and „imaging").

„the discussion is mainly a literature review (normally should be in introduction), the discussion should comment on the authors results with comparision of similar studies from other centers"
Reply: We reworked the discussion part oft he manuscript extensively. The discussion is now structured with the paragraphs Lesions in newborns and infants – Cystic Lesions Lesions in older children – Ovarian torsion Lesions in adolescents – Ovarian Tumours. We present in each paragraph the results of our own observation and discussed it with new sources in the literature, as suggested by the reviewers comment. In addition, we introduced some very recent studies in the literature, as suggested in the comment No 5 by reviewer 1.
(see manuscript discussion part pp. 11 & 12– changes marked in red – paragraphs „Lesions in newborns and infants" – „Cystic Lesions Lesions in older children – „Ovarian torsion" – „Lesions in adolescents – Ovarian Tumours",).

„the conclusion should be based on the authors own results"
Reply: The conclusions were now structured in concordance to the discussion part and now is based on the own results, as suggested by the comment. (see manuscript conclusion part p.14– changes marked in red).

„the term rare but frequent is contradictory."
Reply: The term was changed according to thee referees comment (see manuscript introduction part pp. 3 first line – changes marked in red).

„Most references are rather old"
Reply: We introduced some very recent studies in the literature fort he discussion part, as pointed out above, as suggested in the comment No 5 by reviewer 1. (see reference list, changes marked in red, citations nr. 18, 19, 20, and 21)

Response to reviewer 2

„One of the (main) indication of surgery in newborn girls with a large asymptomatic cyst is to minimise the risc of torsion."
Reply: We stressed this aspect for possible indictation of surgery in the 1st paragraph in our new introduction part according tot he referees comment . (see manuscript introduction part p. 4– changes marked in red – second last sentence).

Reviewers’ Comments to Revised Submission

Reviewer 1: Shehata, Sameh

Date received: 10-Jul-2021
Reviewer recommendation: **Accept in present form**
Reviewer overall scoring: **High**

Assessment Form scores: 5 = High/Yes; 3 = Medium/Adequate; 1 = Low

Is the subject area appropriate for the journal			3		
Does the title clearly reflect the paper's content?		4			
Does the abstract clearly reflect the paper's content	5				
Do the keywords clearly reflect the paper's content?	5				
Does the introduction present the problem clearly?	5				
Are the results/ conclusions justified?		4			
How comprehensive and up-to-date is the subject matter presented?		4			
How adequate is the data presentation?		4			
Are units and terminology used correctly?		4			
Is the number of cases adequate?		4			
Are the experimental methods/ clinical studies adequate?		4			
Is the length appropriate in relation to the content?		4			
Does the reader get new insights from the article?			3		
Please rate the practical significance.		4			
Please rate the accuracy of methods.		4			
Please rate the statistical evaluation and quality control.		4			
Please rate the appropriateness of the figures and tables.	5				
Please rate the appropriateness of the references.		4			
Please evaluate the writing style and use of language.		4			
Please judge the overall scientific quality of the manuscript.		4			
Are the methods used worthy of reproduction in greater deal?	Yes				
Would you be willing to review a revision of this manuscript?	Yes				

Comments to author: The required comments and changes were carefully done by the authors.

Reviewer 2: Kutasy, Balazs

Date received: 24-Jun-2021
Reviewer recommendation: **Accept in present form**
Reviewer overall scoring: **Excellent**

Assessment Form scores: 5 = High/Yes; 3 = Medium/Adequate; 1 = Low

Is the subject area appropriate for the journal	5				
Does the title clearly reflect the paper's content?	5				
Does the abstract clearly reflect the paper's content	5				
Do the keywords clearly reflect the paper's content?	5				
Does the introduction present the problem clearly?	5				
Are the results/ conclusions justified?	5				
How comprehensive and up-to-date is the subject matter presented?	5				
How adequate is the data presentation?	5				
Are units and terminology used correctly?	5				
Is the number of cases adequate?	5				
Are the experimental methods/ clinical studies adequate?	5				
Is the length appropriate in relation to the content?	5				
Does the reader get new insights from the article?	5				
Please rate the practical significance.	5				
Please rate the accuracy of methods.	5				
Please rate the statistical evaluation and quality control.	5				
Please rate the appropriateness of the figures and tables.	5				
Please rate the appropriateness of the references.	5				
Please evaluate the writing style and use of language.	5				
Please judge the overall scientific quality of the manuscript.	5				
Are the methods used worthy of reproduction in greater deal?	Yes				
Would you be willing to review a revision of this manuscript?	Yes				

Comments to author: Much better after modification. Readible, more focused on the topic. Nice review.

Comments by the Editor-in-Chief to Revised Submission

The authors clarified all reviewers comment and questions appropriately.
